# Supplementary material for: Corneal dysfunction precedes the onset of hyperglycemia in a mouse model of diet-induced obesity
Source: PLoS One. 2020 Sep 4;15(9):e0238750. doi: 10.1371/journal.pone.0238750 (PMC7473521; doi:10.1371/journal.pone.0238750)
Supplement: Attachment — Submitted filename: ResponseToReviewers.docx [file pone.0238750.s002.docx]

Re: PONE-D-20-21463, Corneal dysfunction precedes the onset of hyperglycemia in a mouse model of diet-induced obesity

Dear Editor,

Thank you for the review and the opportunity to address the questions and comments. Please find enclosed our responses below. The comments are in bold and our responses are in plain text. Changes to the manuscript are indicated under track changes.  Changes to the excel spreadsheets are as indicated.

We hope the changes to our manuscript are acceptable and that the manuscript is now suitable for publication.

Sincerely,

Aubrey Hargrave and Alan Burns

**Response to Reviewer and Editor Comments:**

**1. “Please present data on systemic changes as a Table (maybe as part of Table 1) and the data for 1 day and 3 days on HFD with quantitation.”**

**“the data on systemic changes in body composition, metabolism and leucocytes were presented only in the text (lines 189-201), whereas the data for 1 day and 3 days on HFD were merely descriptive (lines 190-193) lacking any quantification. They were also absent from the Supplemental Section that contains raw data for Figs 1-7.”**

Both Reviewer #1 and the Editor wanted to see the systemic data for the 10 week mice (described in lines 189-201) reported in tabular form. We have added a new Table 1 (line 203-204) for 10 week HFD systemic changes and updated the text accordingly. We renamed the previous table with 10 day HFD changes to Table 2 and updated the text to reflect this.

We apologize for the confusion in the Supplemental data – the changes in body composition (eAT) and flow cytometry data for the 10 week mice were located in the “Results Text” tab of the excel spreadsheet, along with all other data discussed in the text without a corresponding figure. In order to make these data easier to locate, we have placed it under a separate excel tab (Table 1-10w systemic data).

Thank you for pointing out the discrepancy with the 1 day and 3 day HFD data (line 190-193). We were referring to data from our previously published study and neglected to end the sentence with a citation. The citation has now been added (#57).

**2.** **“The raw data for IL-12p40 and for FACS of leucocyte subsets (presented in the text, need to be incorporated into the Supplemental Section.”**

**“Similarly, the raw data for IL-12p40 (line 247) and for flow cytometric evaluation of leucocyte subsets (presented in the text, lines 267-270) are absent from Supplemental Section.”**

We again apologize for the confusion in the Supplemental data. As noted, raw data for the 10 week cytokines evaluated from whole corneas from line 247 (IL-12p40, as well as IL-1A and IL-10) and the flow cytometry data for the 10 day HFD mice (line 267-270) were previously located together in one “Results Text” tab of the excel spreadsheet. In order to make these data easier to locate, we have placed it into separate excel tabs (Text-10w whole cornea analytes and Text-10d systemic data).

**3. Please consider changing the abbreviation “DR” for diet reversal to something else (like DiR) as it may be confused, especially in this context, with “diabetic retinopathy”.**

We agree with the reviewer and editor, and have made the change from DR to DiR throughout the manuscript, as well as in Fig 2 and Fig 3, to avoid any confusion.

**4. Please ensure that your manuscript meets PLOS ONE's style requirements, including those for file naming.**

We have reviewed the PLOS ONE style templates and believe the manuscript meets the style requirements.

**5. Please amend your current ethics statement to confirm that your named ethics committee specifically approved this study.**

We amended the ethics statement as follows:

“The ethics committees at Baylor College of Medicine Animal Care and Use (IACUC approval #AN2721), and at the University of Houston Animal Care and Use (IACUC approval #16-005) approved this study.”

This sentence was also updated within the Materials and Methods section to read:

“The Animal Care and Use ethics committees at Baylor College of Medicine (IACUC #AN2721), and at the University of Houston (IACUC #16-005) approved this study.”
